# Effect of QTU prolongation on hyperemic instantaneous wave-free ratio value: a prospective single-center study

**DOI:** 10.1007/s00380-020-01562-8

**Published:** 2020-01-27

**Authors:** Masafumi Nakayama, Takashi Uchiyama, Nobuhiro Hijikata, Yuichi Kobori, Nobuhiro Tanaka, Kiyotaka Iwasaki

**Affiliations:** 1 Cardiovascular Center, Todachuo General Hospital, Toda, Japan; 2 Cooperative Major in Advanced Biomedical Sciences, Joint Graduate School of Tokyo Women’s Medical University and Waseda University, Waseda University, 2-2 Wakamatsucho, Shinjuku, Tokyo 162-8480 Japan; 3 Department of Cardiology, Tokyo Medical University Hachiouji Medical Center, Hachiouji, Japan; 4 Department of Modern Mechanical Engineering, Waseda University, Shinjuku, Tokyo Japan

**Keywords:** Coronary artery disease, Coronary circulation, Fractional flow reserve, Instantaneous wave-free ratio, QT prolongation

## Abstract

We hypothesized that in patients with QT prolongation, resistance might not decrease in the wave-free period, because QTU prolongation cannot be detected by instantaneous wave-free ratio (iFR) analysis software. We investigated whether corrected QTU (QTUc) prolongation affects the hyperemic iFR value. Forty-two consecutive patients with intermediate stenosis (≥ 50%) in the left anterior descending coronary artery (LAD) were analyzed. Fractional flow reserve (FFR) and hyperemic iFR were simultaneously and continuously recorded with intravenous adenosine triphosphate (ATP) and papaverine infusions. In 17 patients with stenosis in the proximal LAD, coronary flow was measured. Patients were divided into two groups according to the median absolute deviation of the QTUc by ATP administration/QTUc by papaverine administration. FFR, hyperemic iFR, and flow data were compared between each stimulus and group. Moreover, influences of pressure and electrocardiogram parameters on differences in iFR values under ATP and papaverine administration were compared between the following two groups (group 1: the absolute difference of hyperemic iFR values between ATP and papaverine administration is ≤ 0.05; group 2: that is > 0.05). The paired t test and t test were used in analysis. Hyperemic iFR values of patients under the use of papaverine were lower than those of patients under the use of ATP when QTUc was more prolonged by papaverine administration than by ATP administration (ATP 0.74 ± 0.14, papaverine 0.71 ± 0.15, *P* = 0.025). No significant differences were observed in the FFR value and flow data between the groups. Regarding QTU, QTUc, and QTUc by ATP/QTUc by papaverine, significant differences were observed between group 1 and group 2. Pressure parameters did not induce significant differences. QTUc prolongation induced by papaverine was associated with lower hyperemic iFR values. An iFR-based assessment might lead to inappropriate treatment of patients with QTUc prolongation.

## Introduction

Fractional flow reserve (FFR) is recognized as the primal assessment in determining whether a stable stenotic coronary artery lesion should be interventionally treated [1-6]. Measurement of FFR requires the administration of a vasodilator, e.g., adenosine, adenosine triphosphate (ATP), or papaverine, to produce hyperemia. Then, under minimized flow resistance, the FFR value calculated from the coronary artery distal pressure (Pd) and aortic pressure (Pa) as Pd/Pa over the whole cardiac cycle is used as the index of flow of the lesion [7-9].

Recently, a coronary revascularization strategy guided by the instantaneous wave-free ratio (iFR), which can be measured without the need of administering hyperemic agents, was reported to be noninferior to an FFR-guided revascularization strategy in terms of major adverse cardiac events within 1 year [10, 11]. However, some reports showed a discrepancy in diagnoses based on FFR and iFR in specific clinical and angiographic characteristics [12, 13], and little is known about what factor may induce the discrepancy.

Regarding the FFR-based diagnosis, diastolic FFR has attracted attention for improving accuracy, because high coronary blood flow occurs during diastole in the left anterior descending coronary artery (LAD). Some reports showed that diastolic FFR calculated using electrocardiography or left-ventricular pressure might improve diagnostic accuracy of ischemia in comparison with FFR [14-16]. However, hyperemic iFR has shown no superiority to FFR to date [17]. The iFR value is calculated as the ratio of mean Pd/Pa during the diastolic wave-free period (WFP). In the algorithm of iFR, WFP is identified using aortic pressure waveform. We hypothesized that in patients with QT prolongation after intracoronary papaverine, microvascular resistance might not decrease enough even in the mid-to-end-diastolic phase, because T and U waves represent repolarization of the ventricular muscle [18-20].

The aims of this study were to investigate the impact of QTU prolongation on the iFR value during hyperemia and to better understand the physiological assessment of the severity of coronary artery stenosis. We investigated influences of the corrected QTU (QTUc) prolongation on the hyperemic iFR value using ATP and papaverine.

## Materials and methods

### Study design and subject selection

In this prospective single-center study, 47 consecutive patients who were scheduled for coronary angiography or percutaneous coronary intervention with suspected coronary stenosis in the LAD at Todachuo General Hospital from November 2015 to February 2017 were enrolled. Patients with the presence of more than intermediate stenosis (≥ 50%) in the LAD by angiography and sinus rhythm, and the absence of asthma were included in this study. For those patients, the intake of calcium-channel blockers, coronary vasodilators (dipyridamole, isosorbide mononitrate, isosorbide dinitrate, and nicorandil), theophylline, and caffeine were prohibited for more than 12 h before catheterization. All patients provided written informed consent, and this study was approved by the Institutional Review Board of Todachuo General Hospital. This investigation conformed to the principles outlined in the Declaration of Helsinki.

### iFR and FFR measurements

The Volcano s5 imaging system (iFR version, FFR 2.4.1; Volcano Corporation, San Diego, CA, USA) and Combo Map with the Combo wire or Verrata pressure guide wire (Volcano Corporation) were used for measuring coronary pressure. In addition, in cases where stenosis was identified in the proximal part of the LAD, coronary flow reserve (CFR) was measured at the same time. After the pressure was calibrated to the normal atmosphere before insertion, pressure equalization was performed at the tip of the catheter before advancing it into the distal stenotic lesion. Then, baseline Pd, Pa, and average peak flow velocity (APV) were recorded. FFR and hyperemic iFR were continuously recorded at the same time using iFR scout (Volcano Corporation) with both intravenous ATP and papaverine infusions in all patients. At first, 140 μg/kg/min of ATP was intravenously administered to the patients, and pressures were monitored for at least 3 min or longer. When the Pa and Pd values were confirmed to have returned to their baseline values, FFR, hyperemic iFR, and CFR were measured after intracoronary administration of 12 mg of papaverine to the left coronary artery. The position of the pressure wire was not moved during physiological assessments using two drug stimuli. Hyperemic iFR was measured using fully automated algorithms applied to the WFP in mid-to-late diastole of the cardiac cycle. Pa and Pd values were automatically recorded every 5 ms during the physiological measurements in S5. Pressure waveforms were exported as Excel files (Microsoft Corp., Redmond, WA, USA), and then, FFR values were calculated as Pd/Pa over the cardiac cycle during maximal hyperemia with each stimulus.

CFR was determined by dividing APV at maximal hyperemia for each drug by APV at baseline. The values were calculated automatically by Combo Map. Hyperemic stenosis resistance (HSR) and hyperemic microvascular resistance (HMR) indexes were calculated using the following formula:

HSR: (mean Pa–Pd)/APV at hyperemia; HMR: mean Pd/APV at hyperemia.

### Angiographic analysis

Quantitative coronary angiography was performed using an auto-edge detection method with CMS version 7.1 (Medis, Leiden, the Netherlands). The reference diameter, minimum lumen diameter, and percent diameter stenosis were measured using the external diameter of the catheter as a scaling device.

### Electrocardiogram measurements

The electrocardiogram (ECG) was continuously monitored, and any arrhythmia was recorded during the study. The PQ, RR, and QT intervals were measured at baseline and at maximal hyperemia after ATP and papaverine administration. When the administration induced formation of a T-U wave, the QTU interval was measured. The QT and QTU intervals were measured by tangent methods (usually in the precordial leads, but when inappropriate, in other leads that showed the maximal *U* waves). The QT and QTU intervals were corrected by the Bazett formula [21]. The ECG measurements were performed using AXIOM Sensis HEMO EP128 (Siemens AG, Munich, Germany) or RMC-4000 M (Nihon Koden, Tokyo, Japan). The ECGs were interpreted by two cardiologists. When there was disagreement, the cardiologists discussed the results to reach an agreement.

### Data analysis

Baseline clinical characteristics of patients, including the number and locations of stenotic lesions, were obtained. Hypertension, diabetes mellitus, and dyslipidemia were diagnosed according to guidelines [22-24]. FFR and hyperemic iFR were compared between each drug stimulus. Coronary flow data (APV, CFR, HSR, and HMR) and ECG parameters were compared before and after maximal hyperemia under ATP and papaverine administration. The ratios of the QTUc prolongation (QTUc at maximal hyperemia under ATP (QTCc_a)/QTUc at maximal hyperemia under papaverine (QTCc_p) and differences in the iFR values at maximal hyperemia under ATP and papaverine (iFR_a–iFR_p) were calculated. In the same manner, differences of the FFR values (FFR_a–FFR_p) were calculated. Then, these relationships were compared.

Moreover, we divided the values of iFR_a–iFR_p into three groups as follows: iFR_a–iFR_p <  − 0.05,  − 0.05 ≤ iFR_a–iFR_p ≤ 0.05, or iFR_a–iFR_p > 0.05. Because no lesion showed the value of iFR_a–iFR_p <  − 0.05, influences of pressure and ECG parameters on the values were compared between the comparable group and lower iFR_p group ( − 0.05 ≤ iFR_a–iFR_p ≤ 0.05 and iFR_a–iFR_p > 0.05, respectively).

### Statistical analysis

SPSS software (SPSS 19; IBM Corporation, Chicago, IL, USA) was used for statistical analyses. Values are expressed as mean ± standard deviation. The paired t test was used to compare effects of the drugs on the ECG, pressure, and flow data. Using the median absolute deviation of QTUc_a/QTUc_p, values of iFR_a–iFR_p were compared using the t test. The values of FFR_a–FFR_p were also compared using the t test. ECG and pressure parameters of the comparable group and lower iFR_p group were also compared using the t test. A *p*-value < 0.05 was considered significant.

## Results

### Clinical characteristics

Forty-seven patients agreed to participate in this study. Five patients whose coronary arteries did not have significant stenosis were excluded from the study. Finally, 42 patients were enrolled, and their data were obtained for prespecified analysis.

The clinical characteristics of the 42 patients are shown in Table 1. Patients’ mean age was 70.1 ± 9.9 years, and 32 (76.2%) were men. Laboratory data were normal, and large proportions of patients had hypertension (92.6%), diabetes mellitus (33.3%), and dyslipidemia (81.0%). Some patients were current smokers (26.2%) (Table 1).

**Table 1:** Clinical characteristics

Number of patients (male)	42(32)
Age, years	70.1 ± 9.9
Body weight, kg	64.8 ± 14.4
Body height, cm	161.9 ± 7.7
BMI, kg/m^2^	24.5 ± 3.9
*Laboratory data*	
Hb, g/dL	13.5 ± 1.7
Cr, mg/dL	0.86 ± 0.21
eGFR, ml/min/m^2^	65.7 ± 14.7
Na, meq/L	141 ± 2
K, meq/L	4.3 ± 0.3
Cl, meq/L	104 ± 2
*QCA*	
Lesion length, mm	16.0 ± 8.7
Reference diameter, mm	2.5 ± 0.6
Minimal luminal diameter, mm	1.4 ± 0.5
Diameter stenosis, %	42.4 ± 16.3
Left ventricular ejection fraction, %	64.5 ± 7.8
*Medical history, %*	
Hypertension	39(92.6)
Diabetes mellitus	14(33.3)
Dyslipidemia	34(81.0)
Current Smoking	11(26.2)
Prior myocardial infarction (RCA/LAD/LCX)	8/3/2

*BMI* body mass index, *Hb* hemoglobin, *Cr* creatinine, *eGFR* estimated glomerular filtration rate, *Na* serum sodium, *K* serum potassium *Cl*, serum chloride, *QCA* quantitative coronary angiography, *RCA* right coronary artery, *LAD* left anterior descending artery, *LCX* left circumflex artery

### Hemodynamic and ECG changes after ATP and papaverine infusions

The hemodynamic responses obtained under each drug stimulus are shown in Table 2. There was no significant difference in FFR and hyperemic iFR values between each stimulus (*p* = 0.551 and 0.296, respectively). In 17 patients with stenosis in the proximal part of the LAD, coronary flow was measured, and there was no significant difference in APV, CFR, HSR, and HMR between ATP and papaverine administration. The PQ intervals were significantly shortened after ATP administration at maximal hyperemia. The RR, QTU, and QTUc were significantly prolonged after both papaverine and ATP administration. Furthermore, in comparing the two stimuli, papaverine significantly prolonged QTU and QTUc.

**Table 2:** FFR values, hyperemic iFR values, and ECG parameter in baseline and under the maximal hyperemia by the administration of ATP or papaverine

	Baseline	ATP	Papaverine	*P* value (baseline vs ATP)	*P *value (baseline vs Papaverine)	*P *value (ATP vs papaverine)
Pd, mmHg	87 ± 16	67 ± 14	68 ± 15	< 0.001	< 0.001	0.571
Pa, mmHg	94 ± 15	84 ± 14	84 ± 14	< 0.001	< 0.001	0.756
FFR (Pd/Pa)	0.92 ± 0.05	0.81 ± 0.09	0.81 ± 0.09	< 0.001	< 0.001	0.551
Hyperemic iFR		0.73 ± 0.13	0.73 ± 0.1			0.296
APV, cm/ s (n = 17)	20 ± 6	42 ± 17	43 ± 18			0.297
CFR		2.2 ± 0.7	2.2 ± 0.7			0.269
HSR		0.48 ± 0.25	0.43 ± 0.19			0.300
HMR		1.9 ± 1.0	1.8 ± 0.7			0.343
PQ, msec	162 ± 4	166 ± 26	161 ± 26	< 0.001	0.843	0.274
RR, msec	929 ± 23	854 ± 141	868 ± 143	< 0.001	0.015	0.356
QT, msec	417 ± 8	419 ± 45	408 ± 83	0.003	0.476	0.348
QTU, msec	443 ± 12	470 ± 85	526 ± 75	0.006	< 0.001	< 0.001
QTUc, sec^1/2^	0.467 ± 0.013	0.511 ± 0.013	0.570 ± 0.090	0.002	< 0.001	< 0.001

*ATP* adenosine triphosphate, *Pd* distal coronary pressure, *Pa* aortic pressure, *FFR* fractional flow reserve, *iFR* the instantaneous wave-free ratio, *APV* average peak flow velocity, *CFR* Coronary flow reserve, *HSR* hyperemic stenosis resistance, *HMR* hyperemic microvascular resistance

### Relationship between QTU prolongation and physiological assessment

**Fig. 1:**
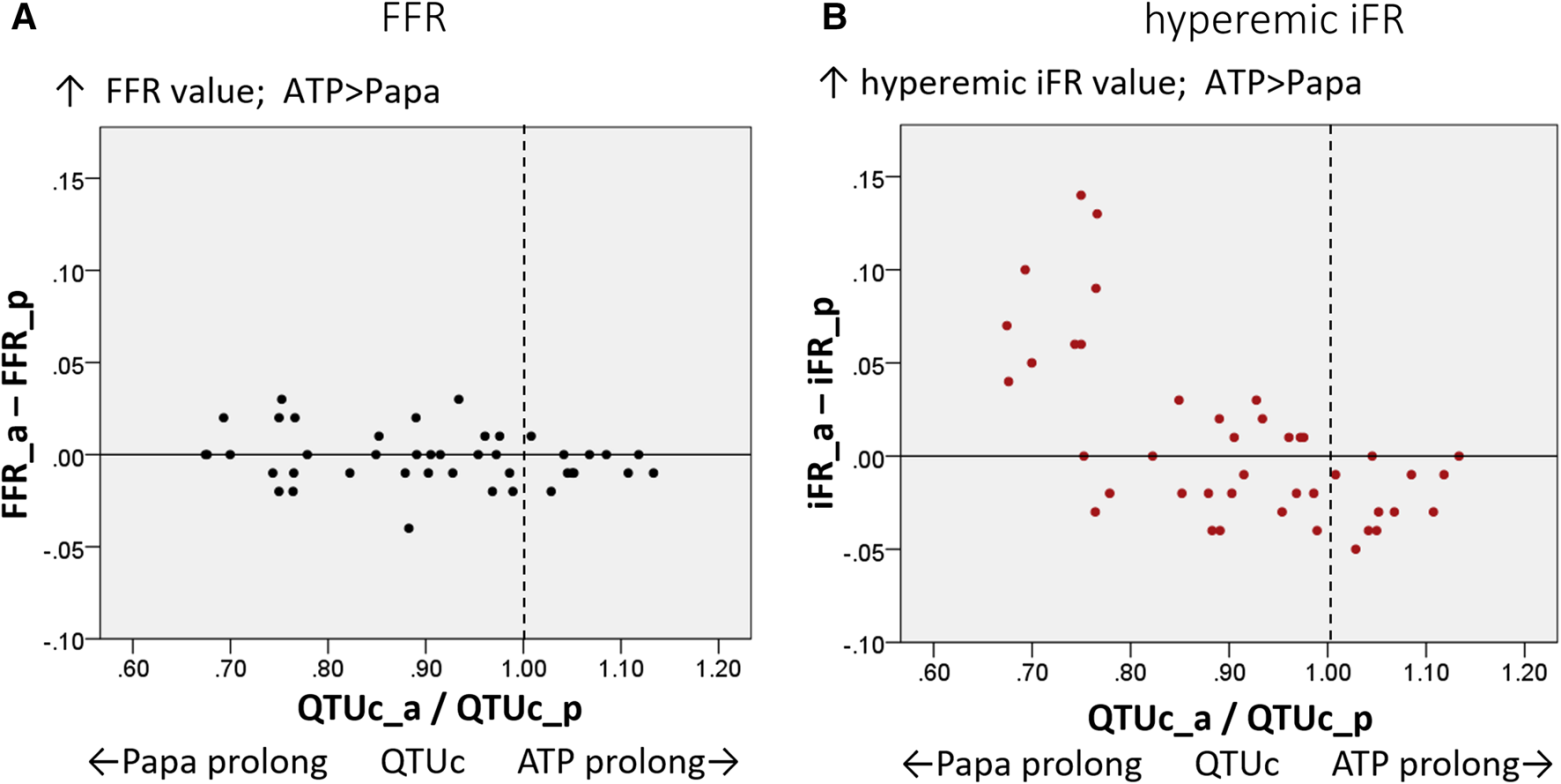
Scatter plot of the relationship between corrected QTC (QTUc) prolongation under hyperemia and fractional flow reserve (FFR) and hyperemic instantaneous wave-free ratio (iFR) values. The relationship between QTUc adenosine triphosphate (ATP)/papaverine ratio (QTUc_a/QTUc_p) and differences of FFR between ATP and papaverine (FFR_a–FFR_p) and differences of hyperemic iFR (iFR_a–iFR_p) are shown in the scatter plot. FFR values under ATP and papaverine administration are equivalent regardless of differences in QTUc values with ATP or papaverine. However, hyperemic iFR values of patients under papaverine administration is lower than those of patients under ATP administration when QTUc was more prolonged by papaverine administration than by ATP administration

The relationship between QTUc ATP/papaverine ratio (QTUc_a/QTUc_p), differences of FFR between ATP and papaverine (FFR_a–FFR_p), and differences of iFR (iFR_a–iFR_p) are shown in the scatter plot (Fig. 1). FFR values under ATP and papaverine administration were equivalent regardless of differences in QTUc_values with ATP or papaverine. However, the hyperemic iFR values of patients under the use of papaverine were lower than those of patients under the use of ATP when QTUc was more prolonged by papaverine administration than by ATP administration. Our study elucidated that hyperemic iFR was affected by QT prolongation, whereas FFR is independent of QT prolongation.

The patients were divided into group 1 and group 2 by the median absolute deviation of QTUc_a/QTUc_p (group 1: 0.674–0.905, group 2: 0.915–1.113). The pressure and flow data after ATP and papaverine administration in each group are shown in Table 3. There was a significant difference between hyperemic iFR in both groups. No significant differences were observed in Pd, Pa, FFR value, APV, CFR, HSR, and HMR between the groups.

**Table 3:** Clinical characteristics, electrocardiogram, pressure, and flow data of patients under the administration of ATP or papaverine between the two groups categorized by the median of QTUc ATP/papaverine ratio

	Group 1 (QTUc ATP/papaverine ratio: 0.674–0.905) (*n* = 21), CFR (*n* = 12)			Group 2 (QTUc ATP/papaverine ratio: 0.915–1.113) (*n* = 21), CFR(*n* = 5)		
Age, years	71.9 ± 8.6			68.4 ± 11.0		
Gender (male %)	15/6 (71.4%)			17/4 (81.0%)		
BMI, kg/m^2^	24.1 ± 4.1			25.0 ± 3.8		
	ATP	Papaverine	*P* value	ATP	Papaverine	*P* value
*Electrocardiogram data*						
PQ interval, msec	172 ± 29	167 ± 28	0.455	159 ± 22	155 ± 24	0.433
RR interval, msec	827 ± 151	819 ± 124	0.729	880 ± 129	916 ± 146	0.080
QT interval, msec	409 ± 46	408 ± 93	0.968	429 ± 42	409 ± 73	0.091
QTU, msec	425 ± 67	535 ± 72	< 0.001	515 ± 78	518 ± 79	0.758
QTUc, sec^1/2^	0.470 ± 0.066	0.594 ± 0.079	< 0.001	0.552 ± 0.084	0.545 ± 0.089	0.454
*Pressure and flow data*						
Pd, mmHg	66 ± 15	66 ± 15	0.862	69 ± 13	71 ± 14	0.365
Pa, mmHg	82 ± 16	81 ± 14	0.754	85 ± 13	86 ± 14	0.500
FFR value	0.81 ± 0.10	0.81 ± 0.10	0.906	0.81 ± 0.09	0.82 ± 0.08	0.217
Hyperemic iFR value	0.74 ± 0.14	0.71 ± 0.15	0.025	0.73 ± 0.12	0.74 ± 0.12	0.009
APV, cm/s	45 ± 17	47 ± 18	0.238	34 ± 15	33 ± 13	0.529
CFR	2.2 ± 0.8	2.3 ± 0.7	0.219	2.0 ± 0.8	2.0 ± 0.7	0.778
HSR	0.46 ± 0.25	0.40 ± 0.19	0.140	0.54 ± 0.26	0.49 ± 0.21	0.233
HMR	1.8 ± 1.1	1.6 ± 0.7	0.271	2.3 ± 0.6	2.3 ± 0.5	0.839

*QTUc* Corrected QTU interval, *BMI* body mass index, *Pd* distal coronary pressure, *Pa* aortic pressure, *FFR* fractional flow reserve, *iFR* the instantaneous wave-free ratio, *APV* average peak flow velocity, *CFR* Coronary flow reserve, *HSR* hyperemic stenosis resistance, *HMR* hyperemic microvascular resistance

**Fig. 2:**
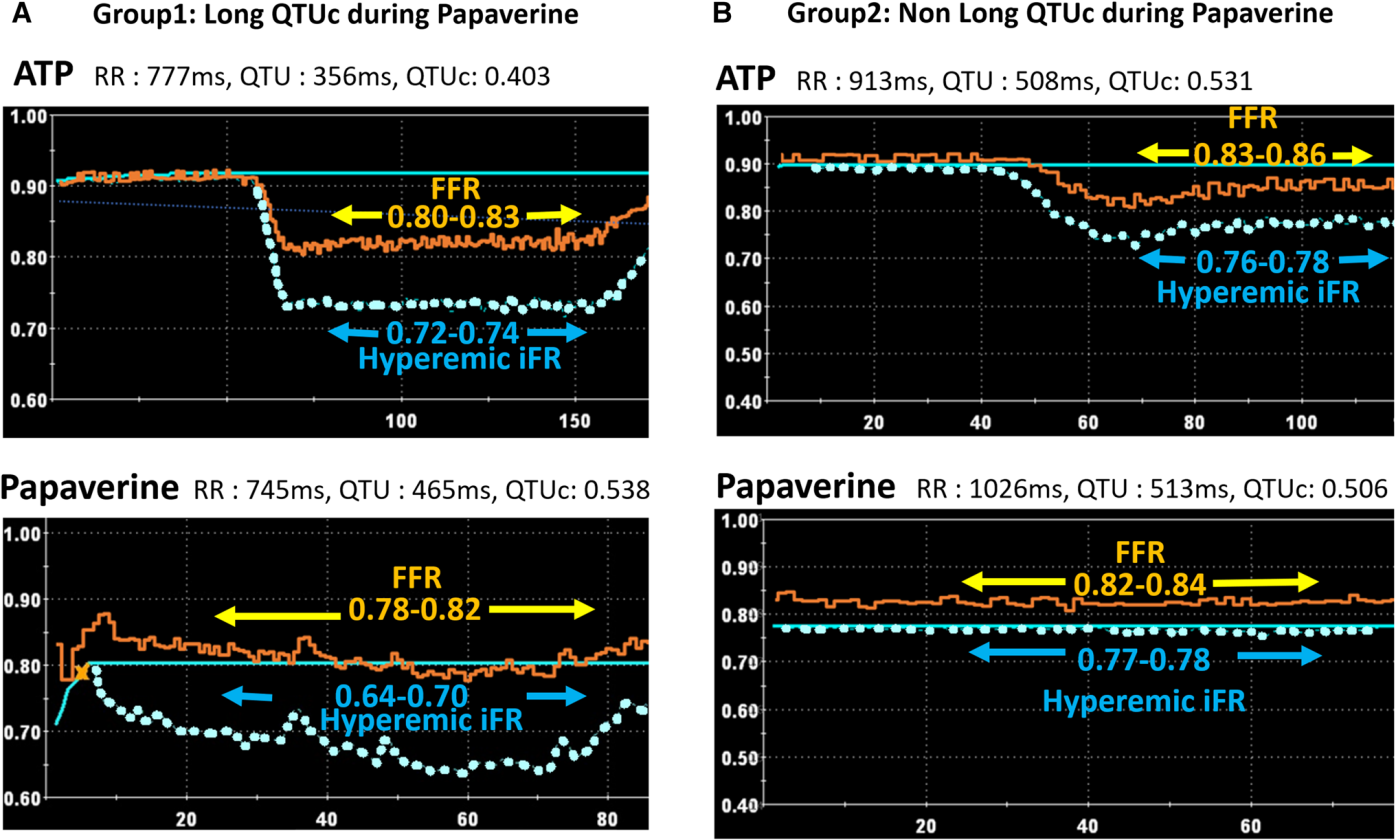
Typical changes in the fractional flow reserve (FFR) and hyperemic instantaneous wave-free ratio (iFR) values under hyperemia by adenosine triphosphate and papaverine administration. The orange line shows the FFR value, and the light blue dotted line shows the hyperemic iFR value. **a** Group 1: long corrected QTU (QTUc) at maximal hyperemia under papaverine administration. Both FFR and hyperemic iFR values were obtained based on steady-state hyperemia by intravenous ATP administration. However, the hyperemic iFR value gradually decreases with fluctuation by papaverine administration. **b** Group 2: non-long QTUc at maximal hyperemia under papaverine administration. Both FFR value and hyperemic iFR values are stable without fluctuation regardless of ATP or papaverine administration when maximal hyperemia was obtained. Furthermore, minimal values of the FFR and hyperemic iFR under two stimuli are comparable

Typical changes in iFR and FFR data during maximal hyperemia of patients in each group are shown in Fig. 2. In patients with QTUc prolongation after papaverine infusion, the iFR value gradually decreased with fluctuation. FFR values were comparable between ATP and papaverine administration (Fig. 2a). In patients without QTUc prolongation after papaverine injection, iFR and FFR values plateaued during maximal hyperemia with both stimuli. Moreover, hyperemic iFR and FFR values were, respectively, comparable between the two stimuli (Fig. 2b).

The difference in FFR values under the two stimuli, i.e., the FFR value under ATP administration minus that under papaverine administration, was not observed between group 1 and group 2 (group 1:  − 0.0005 ± 0.0166, group 2:  − 0.0033 ± 0.0120, *p* = 0.526). However, regarding the difference in iFR values, significantly larger differences were observed in group 1 than in group 2 (group 1: 0.03 ± 0.05, group 2:  − 0.01 ± 0.02, *p* = 0.002).

Seven lesions were assigned to the lower iFR_p group, and 35 lesions were assigned to the comparable group. Regarding QTU interval under ATP administration, QTUc under papaverine administration, and QTUc_a/QTUc_p, significant differences were observed between the two groups. Pressure parameters did not induce significant differences between the two groups (Table 4).

**Table 4:** Influences of pressure and electrocardiogram parameters on the differences in hyperemic iFR values under the administration of ATP and papaverine: comparison between comparable group and lower iFR_p group

	Comparable group − 0.05 < iFR_a–iFR_p < 0.05	Lower iFR_p group 0.05 < iFR_a–iFR_p	*P* value
*n*	35	7	
Age, years old	69.4 ± 9.7	73.7 ± 11.1	0.299
Gender (male %)	26 (74.3%)	6 (85.7%)	0.461
BMI, kg/m^2^	24.4 ± 4.1	24.8 ± 3.0	0.838
ATP			
Pd, mmHg	68 ± 15	64 ± 7	0.504
Pa, mmHg	84 ± 15	81 ± 7	0.696
FFR	0.81 ± 0.09	0.80 ± 0.12	0.657
iFR_a	0.73 ± 0.13	0.74 ± 0.16	0.896
*Papaverine*			
Pa, mmHg	85 ± 15	79 ± 9	0.357
Pd, mmHg	69 ± 15	63 ± 12	0.278
FFR	0.82 ± 0.09	0.79 ± 0.12	0.557
iFR_p	0.74 ± 0.12	0.65 ± 0.18	0.096
*ATP*			
QTU interval, msec	482 ± 85	409 ± 60	0.037
QTUc, sec^1/2^	0.52 ± 0.087	0.461 ± 0.057	0.095
*Papaverine*			
QTU interval, msec	521 ± 72	552 ± 90	0.338
QTUc, sec^1/2^	0.558 ± 0.084	0.629 ± 0.079	0.046
QTUc_a/QTUc_p	0.939 ± 0.117	0.734 ± 0.036	< 0.0001
Lesion length, mm	16.2 ± 9	14.6 ± 7.1	0.650
Reference diameter, mm	2.6 ± 0.6	2.3 ± 0.8	0.263
Minimal luminal diameter, mm	1.45 ± 0.5	1.4 ± 0.5	0.792
Diameter stenosis, %	43 ± 17.2	39.4 ± 10.5	0.599

*ATP* adenosine triphosphate, *Pd* distal coronary pressure, *Pa* aortic pressure, *FFR* fractional flow reserve, *iFR_a* the instantaneous wave-free ratio values at maximal hyperemia under ATP, *iFR_p* the instantaneous wave-free ratio values at maximal hyperemia under papaverine, *QTUc* Corrected QTU interval, *QTUc_a/QTUc_p* The ratios of the QTUc at under ATP / QTUc at under papaverine

## Discussion

FFR and hyperemic iFR values were comparable between ATP and papaverine administration, respectively, in the patients who did not show QTUc prolongation after papaverine administration. However, in patients who showed longer QTUc under papaverine administration than under ATP administration, hyperemic iFR values were significantly lower under the use of papaverine than ATP. FFR values were comparable between ATP and papaverine administration, regardless of QTUc prolongation due to papaverine administration. To our knowledge, this is the first study to show that QTUc prolongation during hyperemia with the use of papaverine distinctly changes hyperemic iFR values. This study revealed that hyperemic iFR is affected by QT prolongation, whereas FFR is independent of QT prolongation. This finding indicates that QT fluctuation could affect iFR under rest.

### Difference in the effect of QTU prolongation on FFR and hyperemic iFR

In this study, we found that hyperemic iFR values were significantly lower under papaverine administration than under ATP administration in patients with prolonged QTUc. The most important difference in algorithm between FFR and iFR is the time of pressure data used for the calculation. The FFR analyzes the whole cardiac cycle, whereas the iFR extracts and calculates only the mid-to-late diastole phase or WFP. iFR values are calculated using the WFP between the beginning 25% of the way into diastole and ending 5 ms before the end of diastole [17, 25]. This period was chosen to reflect the WFP in diastole when the resistance is considered minimal. With the iFR analysis software (version 2.4.1) employed in this study, QTU prolongation cannot be detected. We considered that QTUc prolongation by papaverine administration relatively changes iFR values because of the short analysis period as compared with the FFR values.

### Decrease in hyperemic iFR value due to QTUc prolongation after intracoronary injection of papaverine

The assumption of iFR is that the resistance during WFP is low and stable. The persistent presence of resistance due to prolonged myocardial contraction in WFP will increase iFR values. However, in this study, hyperemic iFR values decreased because of QTUc prolongation after intracoronary injection of papaverine. This finding implies that abnormal myocardial activity during the diastolic phase may limit application of iFR. The data obtained in this study may contribute to improving the diagnosis of ischemic heart disease using iFR.

Recently, it was reported that the assessment by intracoronary electrocardiogram in addition to FFR might be helpful for making a proper diagnosis of infarct-related coronary artery [26]. Further study is needed to improve accuracy and optimize physiology-based assessment of the severity of coronary artery stenosis.

## Limitations

This study has some limitations. First, this study was conducted in a small number of patients. Second, in this study, effects of QTU prolongation on hyperemic iFR were examined under hyperemic conditions. Hyperemic iFR is not commonly established index previously, although a few studies investigated hyperemic iFR value under adenosine administration [17]. Third, it has not been confirmed whether maximal hyperemia is obtained by the administration of each drug. However, a previous study reported that ATP and papaverine have equivalent maximal hyperemic effects [27]. Indeed, in these study subjects, FFR values using ATP and papaverine were equivalent.

## Conclusions

In patients who showed longer QTUc under papaverine administration than under ATP administration, hyperemic iFR values were significantly lower under the use of papaverine than ATP. FFR values were comparable between ATP and papaverine administration, regardless of QTUc prolongation due to papaverine administration. FFR and hyperemic iFR values were comparable between ATP and papaverine administration, respectively, in the patients who did not show QTUc prolongation after papaverine administration. This study revealed that hyperemic iFR was distinctly affected by QT prolongation, whereas FFR was independent of QT prolongation. This finding indicates that an iFR-based assessment might lead to inappropriate treatment of patients with QTUc prolongation.
